# Submilligram Level of Beetle Antifreeze Proteins Minimize Cold-Induced Cell Swelling and Promote Cell Survival

**DOI:** 10.3390/biom12111584

**Published:** 2022-10-28

**Authors:** Keiko Omori, Ignacio Gonzalez, Cindy Nguyen, Shanti N. Raminani, Victor M. Deleon, Pedro Meza, Jose Zamalloa, Rachel G. Perez, Nelson Gonzalez, Hirotake Komatsu, Ismail H. Al-Abdullah, Xin Wen

**Affiliations:** 1 Department of Translational Research and Cellular Therapeutics, Diabetes and Metabolism Research Institute, City of Hope, Duarte, CA 91010, USA; 2 Department of Chemistry and Biochemistry, California State University Los Angeles, Los Angeles, CA 90032, USA

**Keywords:** antifreeze protein, cell swelling, post-hypothermic preservation survival

## Abstract

Hypothermic (cold) preservation is a limiting factor for successful cell and tissue transplantation where cell swelling (edema) usually develops, impairing cell function. University of Wisconsin (UW) solution, a standard cold preservation solution, contains effective components to suppress hypothermia-induced cell swelling. Antifreeze proteins (AFPs) found in many cold-adapted organisms can prevent cold injury of the organisms. Here, the effects of a beetle AFP from *Dendroides canadensis* (DAFP-1) on pancreatic β-cells preservation were first investigated. As low as 500 µg/mL, DAFP-1 significantly minimized INS-1 cell swelling and subsequent cell death during 4 °C preservation in UW solution for up to three days. However, such significant cytoprotection was not observed by an AFP from *Tenebrio molitor* (TmAFP), a structural homologue to DAFP-1 but lacking arginine, at the same levels. The cytoprotective effect of DAFP-1 was further validated with the primary β-cells in the isolated rat pancreatic islets in UW solution. The submilligram level supplement of DAFP-1 to UW solution significantly increased the islet mass recovery after three days of cold preservation followed by rewarming. The protective effects of DAFP-1 in UW solution were discussed at a molecular level. The results indicate the potential of DAFP-1 to enhance cell survival during extended cold preservation.

## 1. Introduction

Hypothermic (or cold) preservation is a widely used short-term storage for cells, tissues, and organs where these biological samples are usually kept at 4 °C in a cold preservation solution designed to minimize or delay hypothermia (or cold) injuries before scheduled transplantation. The components in cold preservation solutions are crucial for the effectiveness of the cold preservation solution. Developed in the 1980s, the University of Wisconsin (UW) solution soon became the standard solution for pancreata preservation and remains the gold standard for the cold preservation of mammalian cells and other abdominal organs and tissues [1]. Compared to the earlier developed preservation solutions, such as Collins solution and Euro-Collins solution (a modified Collins solution), UW solution is, in particular, efficient in suppressing intracellular cell swelling (edema) during cold storage. Specifically, UW solution consists of a high concentration of potassium ion but a low concentration of sodium ion to suppress hypothermia-induced cell swelling and is thus considered an intracellular type of preservation solution. The addition of physiologically inactive and higher molecular weight carbohydrate and anion, namely raffinose, a trisaccharide, and lactobionate, the carboxylate anion of lactobionic acid, as impermeants is another notable advancement in the UW solution to prevent cell edema. Because of its success in hypothermic preservation, UW solution has been an important standard for comparison in the development of solutions and ingredients in cold preservation [2,3]. 

Antifreeze proteins (AFPs) are a unique class of proteins that lower the freezing point of water non-colligatively and inhibit ice growth and recrystallization through binding to the ice surfaces [4,5]. After discovered as glycosylated proteins in Antarctic fishes in the late 1960s [6], AFPs have been found in many other organisms including insects, plants, and bacteria [7,8,9] where insect AFPs usually exhibit superior activity in preventing inoculative freezing and promoting supercooling than AFPs from other sources [10,11]. AFPs have been naturally investigated as potential protectants in cryopreservation [12,13,14,15,16,17]. It was also reported that AFPs can protect cells from hypothermia injury at high concentrations (e.g., 10 mg/mL or higher), which is attributed to the cell membrane protective role of AFPs [18,19,20,21,22,23]. The effects of AFPs in hypothermic storage, however, have not been studied to the same extent as in cryopreservation, and most of the studies focused on fish AFPs [18,19,20,21,22,23]. Recently, the synergistic effect of AFPs and certain co-solutes (referred to as enhancers) is suggested to be vital for the winter survival of many freeze-avoiding organisms [24]. The co-solutes, such as carbohydrates, oxyanions, and nucleosides, usually at a concentration hundreds of times more than that of the AFP, can further enhance the activity of a beetle AFP from *Dendroides canadensis* (DAFP-1) through their interactions mediated by arginines in the AFP [25,26,27,28], while the AFP at a submilligram level can be beneficial to stabilizing the co-solutes under low temperatures [24,29,30]. Interestingly, effective enhancers for DAFP-1 are often found as key components in cold preservation solutions, such as raffinose and lactobionate in UW solution known to prevent cell edema. Taking the above facts into consideration, we propose that the preservation effectiveness of UW solution may be enhanced by a low level of DAFP-1.

The replacement of β-cells by transplantation is a cure in progress for patients with type 1 diabetes (diabetes mellitus). By re-building the insulin control system inside the bodies of patients, this treatment can eventually render patients independent from regular insulin injections [31,32]. However, factors including the severe shortage of the supply of donor pancreata [31] and the poor quality of the donor cells after a short period of cold preservation limit the progress of successful β-cell transplantation [33,34]. Efforts, such as deriving β-cells from other sources [31] and cryopreservation of pancreatic islets for long-term storage [35], have been made in order to relieve the shortage of the supply, yet neither has succeeded as a treatment. Furthermore, pancreatic β-cells are fragile to be stored hypodermically before transplantation, as they are susceptible to hypothermia-induced swelling and consequent death [34]. Because of the effective components in suppressing cell edema, UW solution remains the standard for β-cells cold preservation, while prolonging the cold storage period for viable β-cells through optimizing the cold preservation solution has been considered as an alternative way to relieve the shortage of the donor cells supply [3]. 

In this study, we utilized rat insulinoma cells, INS-1 cells, as a β-cell model to examine the potency of DAFP-1 and another beetle AFP from *Tenebrio molitor* (TmAFP) in enhancing hypothermic preservation effectiveness of UW solution. TmAFP has high sequence identity with DAFP-1 but lacks arginine residues that are important for interacting with the enhancers [10,26]. We then expanded the investigation into rat islet cells, which are the primary pancreatic β-cells. Molecular mechanisms involved in the protective effect of AFP on cold-preserved β-cells injury were also proposed.

## 2. Materials and Methods

### 2.1. Materials

Chemicals were purchased from Sigma-Aldrich (St. Louis, MO, USA) and Fisher Scientific (Pittsburgh, PA, USA) at ACS grade or better and were used as received. Cell culture media and supplements were purchased from Thermo Fisher (Waltham, MA, USA). Bovine serum albumin (BSA, A-7906) was purchased from Sigma-Aldrich (St. Louis, MO, USA) and was used without further purification. Milli-Q water produced from a Synergy water system (Burlington, MA, USA) was used for buffers and solutions preparation. 

### 2.2. Protein Preparation

The expression and purification of the AFP from *D. canandensis* (DAFP-1) and the AFP from *T. molitor* (TmAFP) followed the previously published procedures [26,36]. Briefly, the AFPs were expressed as fusion proteins in *Escherichia coli* Origami B cells, and then, the cells were harvested by centrifugation at 4 °C. After the cells were disrupted, the crude proteins were purified using immobilized metal ion affinity chromatography (IMAC) (Ni-NTA agarose, Qiagen, Germantown, MD, USA). The tags of the AFPs were cleaved off using enterokinase (GenScript, Piscataway, NJ, USA), and then, the resulting proteins were further purified by using IMAC and ion exchange chromatography. Endotoxin in the purified proteins was removed by filtration through Acrodisc Unit with Mustang E membrane (Pall Corporation, Port Washington, NY, USA), yielding endotoxin levels < 0.005 EU/μg protein as tested by the limulus amebocyte lysate (LAL) assay. The AFPs were characterized using SDS-PAGE gel electrophoresis and high-performance liquid chromatography (HPLC) as previously described [16,26,36].

### 2.3. INS-1 Cell Culture and Toxicity Assays for Purified AFPs

INS-1 cells (gifted by Dr. Ian Sweet, the University of Washington, Seattle, WA, USA) were maintained in culture with RPMI 1640 media supplemented with 10% fetal bovine serum, 10 mM HEPES, 2 mM L-glutamine, 1 mM sodium pyruvate, and 25 µM β-mercaptoethanol and 1× antibiotic-antimycotic solution at 37 °C in a humidified atmosphere containing 5% CO_2_. INS-1 cells were harvested using TrypLE solution, and 1.0 × 10^4^ cells/well in a 96-well plate in quadruplicate were plated one day before starting culture with DAFP-1 or TmAFP at a concentration of 0 (control), 60, 250, 500, or 1000 µg/mL in a total of 100 µL media. The same volume of phosphate-buffered saline (PBS) as the volume of the highest concentration of protein was added to the culture media to the control condition. After 24 h of culture at 37 °C in a humidified atmosphere containing 5% CO_2_, the cell viability was tested by the CellTiter-Blue Assay (Promega, Madison, WI, USA) by following the manufacturer’s instructions.

### 2.4. INS-1 Cell Cold Preservation, Viability Assay, and Size Distribution

On the day of the experiment, INS-1 cells were harvested using TrypLE solution, and cells were aliquoted with 1.0 × 10^6^ cells/group in a microcentrifuge tube. INS-1 cells were then resuspended with ice-chilled 250 µL of the UW solution containing DAFP-1 or TmAFP at a concentration of 0, 250, 500, or 1000 µg/mL and immediately cold preserved at 4 °C for up to 72 h. The same volume of PBS as the highest concentration of the protein was added to the UW solution to the vehicle control group, which was labeled as the PBS control group (approximately 7% of total volume depends on the stock protein concentrations). Before and at the end of cold preservation, a 20 µL of cell suspension sample in the UW solution was taken from each group, stained with trypan blue, and the total live cell number and viability were assessed using a Cellometer Auto T4 Cell Counter (Nexcelom Bioscience, Lawrence, MA, USA). The cell recovery rate was calculated as the total live cell number after the cold preservation in each condition divided by the total live cell number before the preservation. To reduce the variability between the experiments, the viability of each group was normalized by the viability of the UW group (control) at each time point and presented as a viability index. The INS-1 cell images and cell sizes of the samples in the control groups and the UW-AFP groups were acquired using a Cellometer Auto T4 Cell Counter (Nexcelom Bioscience, Lawrence, MA, USA) along with the live cell number and viability assessment during the cold preservation. The percent changes in cell size were calculated as the difference of the live cell mean diameter between the post-preservation and pre-preservation divided by the live cell mean diameter of pre-preservation.

### 2.5. Rat Islet Isolation, Cold Preservation, and Rewarming

Rat islets were isolated from female retired breed Lewis (LEW) rat pancreata using our standard procedure [37]. In brief, the pancreata were distended by ductal injections of ice-cold collagenase solution (2.5 mg/mL, Sigma-Aldrich) followed by enzymatic digestion at 37 °C for 10 min. The islets were purified by centrifugation of the digested tissues using Histopaque-1077 (density: 1.077 g/mL, Sigma-Aldrich). The use of animals and animal procedures were approved by the City of Hope/Beckman Research Institute Animal Care and Use Committee. The purified isolated islets were cultured with CMRL 1066, Supplemented, CIT Modification media (Corning, Corning, NY, USA) containing 0.5% human serum albumin, 0.1 μg/mL insulin-like growth factor-1 (Cell Sciences, Newburyport MA, USA), 10 U/mL heparin sodium (Sagent Pharmaceuticals, Schaumburg, IL, USA), referring to the CMRL islet culture media, for one to two days at 27 °C before the start of the experiment. Approximately, 200 IEQ (islet equivalent)/group was aliquoted in a microcentrifuge tube, resuspended with 100 µL of the ice-chilled UW solution with or without DAFP-1 (0, 250, or 500 µg/mL), and cold preserved at 4 °C for up to 72 h. Subsequently, in some experiments, islets were rewarmed/cultured with the CMRL islet culture media in a 24-well suspension plate for 18 h at a 27 °C followed by 2 h at 37 °C incubator in a humidified atmosphere containing 5% CO_2_.

### 2.6. Rat Islet Recovery and Viability Assessment

Islet mass was determined by taking pictures before and after cold preservation, which was followed by rewarming using an IXDP50 Olympus microscope with a DP74 camera (Olympus America, Center Valley, PA, USA). The area of islets in each image was measured using Olympus CellSens software (Olympus America). The islet mass recovery was calculated as the total islet area at the end of the rewarming period divided by the total islet area before the cold preservation in the corresponding sample as described [38].

Islet viability was assessed before and after cold preservation or after rewarming by 10 µg/mL propidium iodide (PI) and 0.48 µM fluorescein diacetate (FDA) staining under the IXDP50 Olympus microscope. The image of islet viability staining was captured, and the viability was determined as the area of PI staining divided by the area of FDA staining in the corresponding islet as previously described [39].

### 2.7. Glucose-Stimulated Insulin Secretion (GSIS) Assay

Cold-preserved islets (approximately 60 IEQ/sample) were rewarmed with RPMI 1640 media containing 10 mM HEPES, 10% fetal bovine serum (FBS), and 3 mM glucose for 4 h at a 37 °C incubator before the assay. Islets were then placed in a 24-well plate with 1.0 mL of 2.8 mM glucose-Krebs Ringer Buffer (KRB) solution for 60 min followed by 28 mM glucose-KRB solution for 60 min. Following incubation, a sample of the KRB solution was collected, and insulin release was measured by a High Range Rat Insulin ELISA kit (Mercodia, Winston Salem, NC, USA). The stimulation index was calculated as the insulin release during the incubation in the 28 mM KRB solution divided by the insulin release in 2.8 mM KRB solution of the corresponding sample. 

### 2.8. Western Blotting

The total cell lysate from the pre- and post-cold preserved INS-1 cells (3 × 10^6^ cells/group) with or without 500 µg/mL DAFP-1 in the UW solution was collected using the RIPA Lysis and Extraction Buffer (Thermo Fisher, Waltham, MA, USA) containing cOmplete^TM^, EDTA-free Protease Inhibitor Cocktail (Sigma-Aldrich). The protein concentration of the samples was measured by the Quick Start™ Bradford protein assay (Bio-Rad Laboratories Inc., Hercules, CA, USA). A total of 20 µg of protein per sample was mixed with 2× Laemmli sample buffer and separated by SDS-PAGE using 4–20% Tris-Glycine gel (Thermo Fisher), and Western blotting was performed using antibodies against PARP, β-Actin antibody, anti-rabbit IgG horseradish peroxidase (HRP)-linked antibody with LumiGLO reagent (Cell Signaling Technology, Danvers, MA, USA) as previously described [40]. The images were captured by Azure 600 (Azure Biosystems, Dublin, CA, USA) and quantified using CellSens software (Olympus).

### 2.9. Statistical Analysis

Data are reported as mean ± standard error unless specified. Statistical significance was determined using a Student’s *t*-test or one-way ANOVA. Correlations between two parameters were determined using Pearson’s coefficient (r) for parametric comparison. *p*-value less than 0.05 was considered significant.

## 3. Results

### 3.1. The Size and Viability of INS-1 Cells Are Inversely Correlated after Cold Preservation in the Different Preservation Solutions

To confirm the effectiveness of UW solution in preventing cell swelling and preserving cell viability, we first examined the INS-1 cell size and the viability after cold preservation in different solutions, including PBS, PBS(+), that is PBS containing Ca^2+^ and Mg^2+^, or UW solution for 24, 48 or 72 h. After 24 h of storage at 4 °C, the sizes of the INS-1 cells stored in PBS or PBS(+) appear to be larger compared to those preserved in the UW solution with decreased viability (Supplemental Figure S1A–E), indicating the effectiveness of UW solution in suppressing the cells swelling and maintaining the viability during cold preservation. To determine the correlation between the viability and cold-preserved live cell size, the cell viability and cell size measurements at 0 h, 24 h, 48 h, and 72 h cold preservation in each solution group were pooled for the analysis. A significant inverse correlation between the cell size and cell viability was identified (r = −0.73, r^2^ = 0.53, *p* < 0.0001, N = 60) (Supplemental Figure S1F).

### 3.2. The Beetle AFPs Have No Cytotoxicity on INS-1 Cells

To explore whether the beetle AFPs, DAFP-1 and TmAFP, have any detrimental effects on INS-1 cells, we evaluated the potential cytotoxicity of each of the beetle AFPs on INS-1 cell culture. A series of low-level concentrations of the beetle AFPs, including 60, 250, 500, and 1000 µg/mL, were tested in this study. Compared to the INS-1 cell culture without the AFPs, the inclusion of DAFP-1 or TmAFP at all the testing concentrations did not cause apparent toxicity effects on the cell growth/viability of INS-1 cells. Furthermore, there is no significant difference in cell growth/viability between the different concentrations (60, 250, 500, or 1000 µg/mL) of either beetle AFP during a 24 h culture of INS-1 cells (Supplemental Figure S2A,B). These results suggest that at or below 1000 µg/mL, the presence of DAFP-1 or TmAFP does not have cytotoxic effects on the INS-1 cells.

### 3.3. DAFP-1 Vehicle Has No Effect on INS-1 Cell Survival during Cold Preservation in UW Solution

**Figure 1:**
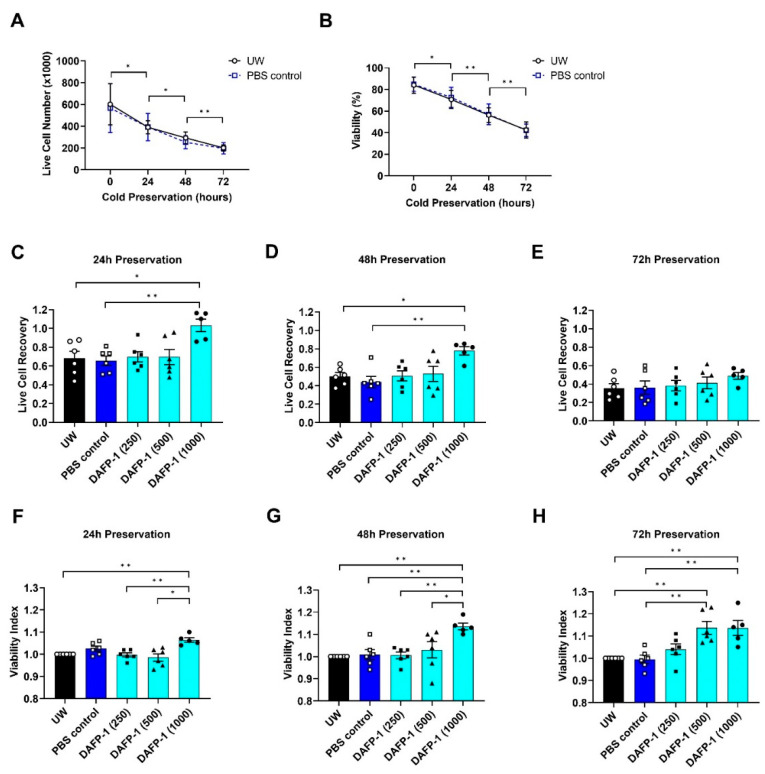
Survival of INS-1 cells cold preserved in UW solution supplemented with DAFP-1. INS-1 cells were cold preserved in UW solution alone, UW solution containing vehicle (PBS control), or with 250, 500, and 1000 µg/mL DAFP-1 for up to 72 h. (**A**) The live cell number and (**B**) the viability of INS-1 cells before and after the cold preservation in the UW solution or PBS control group tested at 24, 48, and 72 h of preservation. The data show the mean ± standard deviation of six independent experiments, * *p* < 0.05, ** *p* < 0.01, by the one-way ANOVA. The live cell number of INS-1 cells after (**C**) 24 h, (**D**) 48 h, and (**E**) 72 h of cold preservation was compared to pre-preservation shown by the live cell recovery (live cell number at each time point divided by the live cell number before preservation), and the INS-1 cell viability of each protein group was compared to the UW solution group at (**F**) 24 h, (**G**) 48 h, and (**H**) 72 h shown as viability index (viability of INS-1 cells preserved in each condition divided by the viability of INS-1 cells preserved in the UW solution). The data show the mean ± standard error of five to six independent experiments (open circle for UW alone, open square for PBS control, solid square, solid triangle, and solid circle for 250, 500 and 1000 µg/mL DAFP-1, respectively), * *p* < 0.05 and ** *p* < 0.01 by the one-way ANOVA.

The effectiveness of UW solution on cold preservation of INS-1 cells at 4 °C was evaluated for up to 72 h. The live cell number of INS-1 cells preserved in UW solution was decreased every 24 h (Figure 1A). There was a 13.9 ± 0.5% reduction in INS-1 cell viability every 24 h cold preserved in UW solution, and after 72 h, the cell viability of the INS-1 cells was reduced to below 50% (Figure 1B). The results suggest that UW solution provides a limited level of protection to the INS-1 cells during the extended period of hypothermic preservation. The same trend was observed in the vehicle control group, PBS added to UW solution (PBS Control), and there was little difference in live cell number and viability between the UW solution group and the PBS control group (Figure 1A,B). In particular, the viability of the vehicle control group was almost identical to that of the UW solution group at each time point. The results suggest that the addition of the vehicle PBS up to 7.0% (*v*/*v*) in UW solution has no effect on the survival or viability of INS-1 cells during hypothermic preservation. The INS-1 cell viability of each protein group was compared to the UW solution group at (F) 24 h, (G) 48 h, and (H) 72 h shown as viability index (viability of INS-1 cells preserved in each condition divided by the viability of INS-1 cells preserved in the UW solution). The data show the mean ± standard error of five to six independent experiments, * *p* < 0.05, ** *p* < 0.01, and *** *p* < 0.001 by the one-way ANOVA.

### 3.4. DAFP-1 Improves INS-1 Live Cell Recovery during Cold Preservation in UW Solution

To evaluate the effect of DAFP-1 on hypothermia preservation, INS-1 cells were cold preserved in UW solution supplemented with or without DAFP-1. Based on the toxicity testing results (Supplemental Figure S2) and the pilot experiment of the cold preservation, the concentrations of the DAFP-1 at 0, 250, 500, and 1000 µg/mL were used in the subsequent 72 h cold preservation studies. 

After 24 h of cold preservation at 4 °C, the live cell recovery decreased in the UW solution group without supplements (0.68 ± 0.07) (Figure 1C) or the PBS control group (0.66 ± 0.05) (Figure 1C), representing over 30% cell death, while similar live cell recovery was also observed in the UW solution supplemented with DAFP-1 at 250 or 500 µg/mL (Figure 1C), suggesting that the supplementation of DAFP-1 at lower concentrations in UW solution has little effect in improving live cell recovery during 24 h of hypothermic preservation. In contrast, the supplementation of DAFP-1 at 1000 µg/mL in UW solution maintained cell survival during 24 h cold preservation as compared to that of the UW solution alone or the PBS control group (Figure 1C). The results indicate that the supplementation of DAFP-1 at 1000 µg/mL in UW solution significantly improves live cell recovery during 24 h of cold preservation at 4 °C (Figure 1C).

A similar trend was observed in the INS-1 live cell recovery for 48 h hypothermic preservation. The supplementation of DAFP-1 at 1000 µg/mL in UW solution significantly increased the live cell recovery as compared to that of the UW solution alone or the PBS control group (0.78 ± 0.04 by DAFP-1 at 1000 µg/mL vs. 0.50 ± 0.04 by UW or 0.44 ± 0.06 by PBS control). The presence of DAFP-1 at 250 or 500 µg/mL shows similar live cell recovery as compared to that of either of the control groups (Figure 1D). The results suggest that the supplement of DAFP-1 at 1000 µg/mL significantly improves live cell recovery during 48 h of cold preservation at 4 °C in UW solution, while such an improvement is not apparent in supplementing at a lower concentration. 

After 72 h of preservation at 4 °C, the live cell recovery of the INS-1 cells dropped to 0.36 ± 0.05 in the UW control group or 0.36 ± 0.07 in the PBS control group (Figure 1E). The UW solution supplemented with 250 µg/mL DAFP-1 shows similar live cell recovery as compared to that of either of the control groups. The live cell recovery of the INS-1 cells in UW solutions supplemented with DAFP-1 at 500 or 1000 µg/mL was 0.41 ± 0.06 or 0.49 ± 0.03, respectively. The results indicate that the presence of DAFP-1 at 500 or 1000 µg/mL improves the live cell recovery during 72 h of cold preservation at 4 °C in UW solution. While the trend was observed, the difference in the live cell recovery between the control group (UW or PBS) and the DAFP-1 group (DAFP-1 at 500 or 1000 µg/mL) was not statistically significant due to the variability between the experiments. 

### 3.5. DAFP-1 Improves INS-1 Cell Viability during Cold Preservation in UW Solution

To discern the effect of DAFP-1 on the cold-preserved INS-1 cells in UW solution, the viability of each group was normalized by the viability of the UW group (control) at each time point and presented as a viability index to reduce the variability between the experiments. The effect of DAFP-1 on hypothermia preservation of INS-1 cells was then further evaluated by comparing the cell viability under each condition with the UW solution group presented as a viability index at each time point (Figure 1F–H).

During the entire course of hypothermic preservation, the viability index of INS-1 cells cold preserved in UW solution supplemented with 1000 µg/mL DAFP-1 was significantly greater than that of either of the control groups (UW or PBS control) (Figure 1F,H). Furthermore, the presence of DAFP-1 at 1000 µg/mL in UW solution resulted in the highest INS-1 cell viability index among all the conditions tested after 24 h and 48 h of cold preservation (Figure 1F,G).

After 24 h or 48 h of cold preservation, the viability index of INS-1 cells in UW solution supplemented with 250 or 500 µg/mL DAFP-1 was similar to that in either of the control group (Figure 1F,G).

After 72 h of preservation, the viability index of the INS-1 cells in UW solution supplemented with 500 µg/mL DAFP-1 was significantly increased to a level (1.14 ± 0.03) (Figure 1H) that is virtually the same as the viability index of the INS-1 cells in UW solution supplemented with 1000 µg/mL DAFP-1 (1.14 ± 0.03) (Figure 1H). These results suggest that DAFP-1 at 500 µg/mL enhances the effectiveness of UW solution toward the long-term hypothermic preservation of INS-1 cells; however, its enhancement effect was not noticeable for the shorter-term hypothermic preservation of INS-1 cells. Similar to that of the group of UW solution supplemented with 500 µg/mL, the presence of DAFP-1 at 250 µg/mL in UW solution also resulted in a noticeable increase in the viability index of INS-1 cells after 72 h of preservation as compared to that in the UW or PBS control group (Figure 1H). These results suggest that the effectiveness of UW solution in hypothermic preservation of INS-1 cells can be enhanced by the beetle AFP at the submilligram level. Collectively, the results shown in Figure 1 suggest that the enhancement effect by the beetle AFP is dose- and duration-dependent.

### 3.6. DAFP-1 Suppresses INS-1 Cell Swelling and Is Correlated with Increased Viability during Cold Preservation

**Figure 2:**
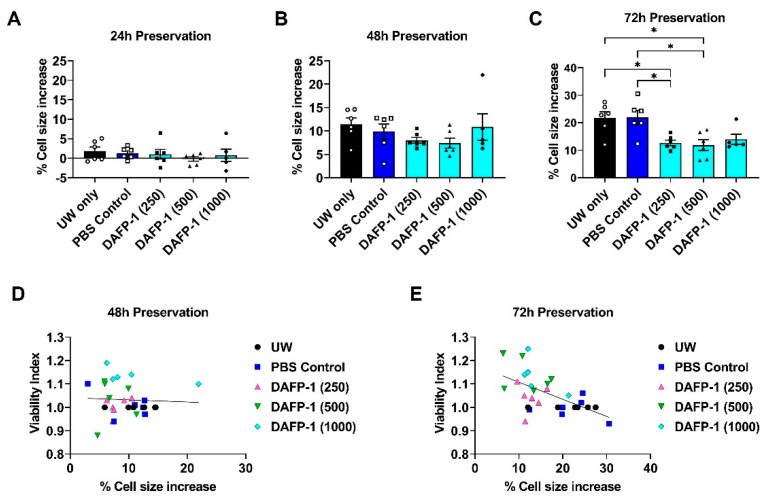
Changes in the size of INS-1 cells cold preserved in the UW solution supplemented with DAFP-1. The INS-1 cells were cold preserved in UW alone, UW solution containing vehicle (PBS control) or with 250, 500, and 1000 µg/mL DAFP-1 for up to 72 h, and the live cell sizes were analyzed along with the cell viability using the trypan blue staining. The percent increases of the live cell size before- and post-cold preservation was calculated as the difference of the mean diameter of the live cells between the post-preservation and pre-preservation divided by that of the pre-preservation at (**A**) 24 h, (**B**) 48 h, and (**C**) 72 h after cold preservation (open circle for UW only, open square for PBS control, solid square, solid triangle and solid circle for DAFP-1 250, 500 and 1000 µg/mL, respectively). The data show the mean ± standard error of five to six independent experiments, * *p* < 0.05 by the one-way ANOVA. (**D**) The overall correlation between the percentage of cell size increase and the viability index of the corresponding samples from all the groups at 48 h preservation and (**E**) 72 h preservation (r = −0.57, r^2^ = 0.33, *p* < 0.005, N = 5 independent experiments with 29 data points).

To determine if the effect of DAFP-1 on INS-1 cell survival was associated with cell swelling, the live cell size of the cold-preserved INS-1 cells in UW solution with or without DAFP-1 was first examined. The live cell size did not change dramatically after 24 h of cold preservation at 4 °C. The percentages of the live cell size increase were within 2% regardless of the preservation solutions used (Figure 2A). The size of the live INS-1 cells increased by about 12% after 48 h of cold preservation in UW solution. There was a trend that the live cell size increase was less when the INS-1 cells were preserved in UW solution supplemented with submilligram levels of DAFP-1 compared to UW solution without DAFP-1 (Figure 2B). Moreover, submilligram levels of DAFP-1 supplements (i.e., 250 and 500 μg/mL) seem to be equally effective if not better when compared to the DAFP-1 supplement at a higher level (i.e., 1 mg/mL) in suppressing the cell swelling after 48 h of cold preservation in UW solution (Figure 2B). This trend was also observed for the submilligram levels of DAFP-1 supplements after 72 h of cold preservation in UW solution (Figure 2C). We thus speculate that the mechanisms of AFPs at submilligram levels may not be the same as they are at milligram levels. The size of the live INS-1 cells increased about 22% after 72 h of cold preservation in UW solution as compared to the pre-preservation, that is, another 10% increase in the cell sizes after additional 24 h of cold preservation (vs. 48 h). In contrast, the live cell size increased by 14% or less as compared to the pre-preservation and by 4% after additional 24 h of cold preservation (vs. 48 h) when preserved in UW solution supplemented with DAFP-1 at all the concentrations (Figure 2C). Notably, the cell size change after 72 h of cold preservation in UW solution with submilligram levels of DAFP-1 was similar to the cell size change after 48 h of cold preservation in UW solution without DAFP-1 (Figure 2B,C). We further analyzed the association between the cell size and the viability changes (% pre-preservation) during cold preservation. There was a significant, strong inverse correlation between the live INS-1 cell size and the viability changes at 72 h of cold preservation (r = −0.57, r^2^ = 0.33, *p* < 0.005, N = 5 independent experiments with 29 data points), while there was no significance at 48 h of cold preservation (Figure 2D,E). The results suggest that the supplementation of UW solution with DAFP-1 further suppresses the cell swelling and improves the effectiveness of UW solution in preserving cell viability during extended cold preservation.

### 3.7. DAFP-1 Reduces Cold Preservation-Mediated INS-1 Cell Apoptosis

**Figure 3:**
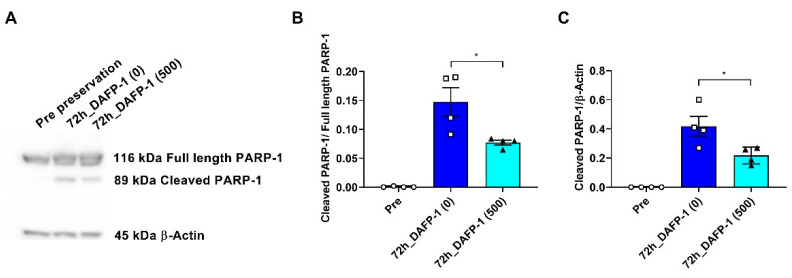
Fragments of poly(ADP-ribose) polymerase-1 (PARP-1) in the cold-preserved INS-1 cells. The total length and cleaved PARP-1 protein expressions in the INS-1 cells before and after 72 h of cold preservation with or without 500 µg/mL DAFP-1 were assessed by Western blot. (**A**) Representative image of the Western blot bands for the total length PARP-1, cleaved PARP-1, and β-actin, (**B**) quantified relative protein expression levels for the cleaved PARP-1 over β-actin, and (**C**) cleaved PARP-1 over total length PARP-1 (open circle for pre preservation, open square for 0 µg/mL, and solid triangle for 500 µg/mL DAFP-1). The data show the mean ± standard error of five independent experiments, * *p* < 0.05 by the Student *t*-test.

Poly(ADP-ribose) polymerase (PARP-1) is a critical enzyme involved in DNA repair, and cleaved PARP has been used as an indication of apoptosis induced by hypothermic injury during cold preservation [41]. The combined results of live cell recovery, cell viability, and cell sizes indicated that DAFP-1 at 500 µg/mL is an optimal concentration for 72 h of cold preservation of INS-1 cells (Figure 1 and Figure 2). We thus chose DAFP-1 at 500 µg/mL to evaluate the molecular mechanism involved in the submilligram level of AFP-mediated hypothermic protection in UW solution through the cleavage of PARP-1 in the cold-preserved INS-1 cells examined by the Western blot. The band densities of the cleaved PARP-1 fragments were normalized to that of the full-length PARP-1 and β-actin from total cell extracts, respectively. Full-length PARP-1 at 116 kDa and an unnoticeable amount of cleaved PARP-1 were observed in the cell extracts of pre-preserved INS-1 cells, while a significant amount of cleaved PARP-1 fragment at 89 kDa, a hallmark of apoptosis, was observed in the extracts of the cells after 72 h hypothermic preservation in UW solution (Figure 3). The amount of cleaved PARP-1 was significantly reduced in the cell extracts after 72 h of hypothermic preservation in UW solution supplemented with 500 µg/mL DAFP-1 (Figure 3). The results suggest that the cold preservation of INS-1 cells induces cell apoptosis, and the supplementation of DAFP-1 at the submilligram level in UW solution reduces the apoptosis process, further validating the enhanced cytoprotection by the presence of submilligram level of DAFP-1 during the cold preservation in UW solution.

### 3.8. DAFP-1 Possesses Higher Potency than TmAFP in Preserving Viable INS-1 Cells in UW Solution

**Figure 4:**
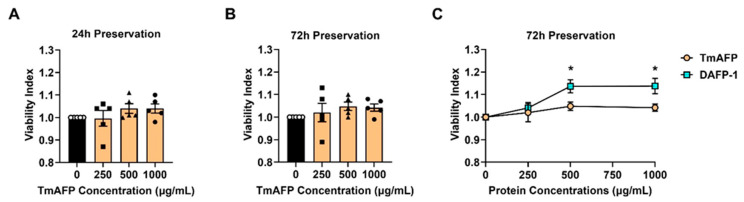
Potency of DAFP-1 in maintaining cold-preserved INS-1 cell viability compared to TmAFP and BSA. The viability index of the cold-preserved INS-1 cells in UW solution supplemented with TmAFP at a different concentration (0, 250, 500, and 1000 µg/mL) for (**A**) 24 h and (**B**) 72 h. The cell viability of each protein condition was normalized by the viability of the UW solution group (AFP 0 µg/mL, open circle) at each time point (solid square, solid triangle, and solid circle for 250, 500, and 1000 µg/mL DAFP-1, respectively). (**C**) The dose-dependent changes of viability indexes for TmAFP and DAFP-1 at 72 h. The data show the mean ± standard error of five to six independent experiments, * *p* < 0.05 by the Student *t*-test.

To evaluate the effects of TmAFP on INS-1 cell cold preservation in UW solution, similar experiments were performed using TmAFP at concentrations of 0, 250, 500, and 1000 µg/mL for up to 72 h. Similar to those in Figure 1, the Viability Index was also used here to compare the potency of TmAFP and DAFP-1 as it eliminates the variations of the initial cell number and viability between the experiments and is considered to be a more sensitive indicator comparing to the live cell recovery presented in Figure 4. When compared to the UW solution group, the INS-1 cell viability was improved to a certain extent in UW solution supplemented with TmAFP at 500 µg/mL or 1000 µg/mL at 24 h and 72 h of preservation, while UW solution supplemented with TmAFP at 250 µg/mL did not show this trend (Figure 4A,B). However, this cytoprotective effect of TmAFP at 500 µg/mL or 1000 µg/mL did not reach statistical significance. The results suggest that the addition of TmAFP at 1000 µg/mL or less provides a minimal improvement in INS-1 cell survival during the 72 h of cold preservation in UW solution. To compare the potency of these two AFPs, the viability index data in Figure 4A,B for the INS-1 cells preserved in UW solution with TmAFP and in Figure 1F–H for the INS-1 cells preserved in UW solution with DAFP-1 are plotted together in Figure 4C. The viability indexes of DAFP-1 at both 500 µg/mL and 1000 µg/mL after 72 h of cold preservation were significantly higher than those of TmAFP; however, there was no significant difference between the samples dosed with the two AFPs after 24 h of cold preservation (Figure 4C). The results suggest that DAFP-1 possesses a higher potency than TmAFP in maintaining cell survival during the extended hypothermia preservation of INS-1 cells in UW solution.

### 3.9. DAFP-1 Improves the Survival of Rat Islets after 72 h of Cold Preservation, Followed by Rewarming

**Figure 5:**
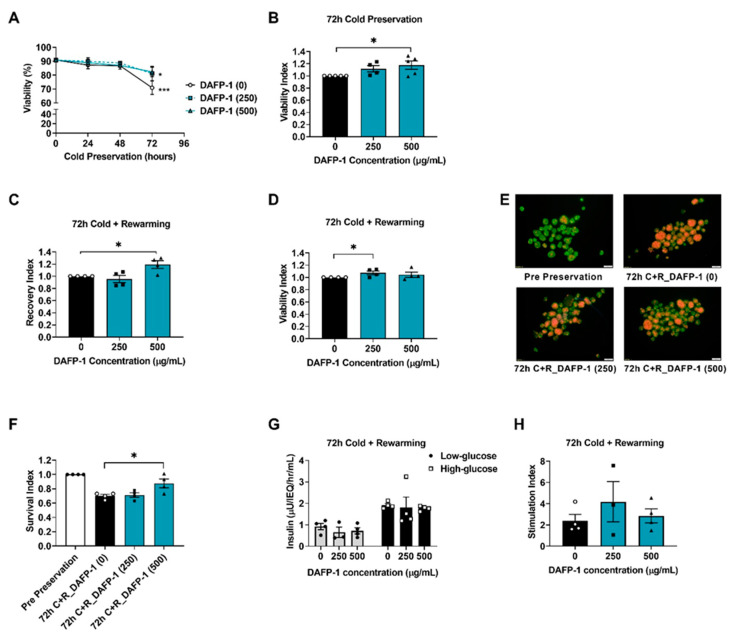
Viability, survival, and function of cold-preserved rat islets. Isolated rat islets were cold preserved in UW solution at 4 °C with or without DAFP-1 (0, 250, or 500 µg/mL) for up to 72 h. (**A**) The changes in cold-preserved islet viability measured by the fluorescent diacetate (FDA) and propidium iodide (PI) staining, and (**B**) Viability Index of each protein condition (solid square for 250 µg/mL DAFP-1 and solid triangle for 500 µg/mL DAFP-1) calculated relative to the control (DAFP-1 0 µg/mL = UW solution alone, open circle). In the separate experiment, rat islets were cold preserved in UW solution with or without DAFP-1 (0, 250, or 500 µg/mL) for 72 h, followed by overnight rewarming in the culture at 27 °C. (**C**) The recovery index and (**D**) viability index of rewarmed islets was shown as relative to the control at each time point. (**E**) The representative images of the 72 h cold-preserved and rewarmed (72 h C + R) rat islet viability assessed with FDA (alive in green)/PI (dead in orange) staining. Scale bar: 200 µm. (**F**) Overall survival after the 72 h cold preservation followed by the rewarming was calculated by the recovery index multiplied by the viability (%) relative to the pre-preservation (solid circle). (**G**) The function of rat islets after 72 h of cold preservation followed by rewarming assessed by the glucose-stimulated insulin release in a static incubation assay (1 h incubation assay in 2.8 mM glucose followed by 1 h incubation in 28 mM glucose) and (**H**) stimulation index calculated by the insulin release during the 28 mM glucose incubation divided by the insulin release during the 2.8 mM glucose incubation. The data show the mean ± standard error of three to four independent experiments, * *p* < 0.05, *** *p* < 0.005 by the Student’s *t*-test.

We have demonstrated that UW solution supplemented with DAFP-1 at 500 µg/mL improves the survival of INS-1 cells after 72 h of cold preservation. Here, we further evaluated the feasibility of this preservation method on the primary pancreatic β cells. In particular, the isolated rat islet, a cluster of 2000–3000 cells with 60–80% of insulin-producing β cells [42], is utilized in this experiment. The islet viability was maintained during the first 48 h but significantly decreased at 72 h of cold preservation in UW solution compared to pre-preservation (70.9 ± 4.8% vs. 90.9 ± 1.0%, respectively, *p* < 0.005). The loss of islet viability after 72 h of cold preservation was less extensive when UW solution was supplemented with submilligram of DAFP-1 at 250 µg/mL and 500 µg/mL (81.1 ± 5.2% and 82.3 ± 3.4%, respectively, *p* < 0.05 vs. pre-preservation) (Figure 5A). The viability index of rat islets after 72 h of cold preservation in UW solution supplemented with 500 µg/mL DAFP-1 (1.17 ± 0.08) was significantly higher than islets preserved in UW solution alone control (Figure 5B). It is known that the cold preservation-mediated islet injury is manifested during the rewarming of the islets [34,43]. Therefore, we further assessed the recovery and viability of islets after cold preservation followed by rewarming. After the 72 h of cold preservation followed by overnight rewarming, the supplementation of UW solution with DAFP-1 at 500 µg/mL significantly increased the recovery index (1.2 ± 0.1, *p* < 0.05 vs. UW) (Figure 5C), while DAFP-1 at 250 µg/mL significantly increased the viability index (1.07 ± 0.02, *p* < 0.05 vs. UW) as compared to UW solution alone (Figure 5D,E). After rewarming, the overall survival of islets preserved in UW solution or with DAFP-1 250 µg/mL significantly decreased as compared to pre-preservation (0.7 ± 0.02 and 0.71 ± 0.03 respectively, *p* < 0.001 vs. pre-preservation), while islets preserved in UW solution with 500 µg/mL DAFP-1 maintained the survival index as compared to pre-preservation (0.87 ± 0.05, *p* = 0.09 vs. pre-preservation) and was significantly higher than UW solution without DAFP-1 group. The supplementation of UW solution with DAFP-1 did not affect the 72 h cold-preserved islet function (Figure 5G,H). The results with rat islet cold preservation further validate the cytoprotective effect of the submilligram level of DAFP-1, 500 µg/mL, to prevent cell death during the 72 h of cold preservation.

## 4. Discussion

UW solution is a current standard intracellular solution for organ preservation. In particular, the higher molecular weight cell membrane impermeants, lactobionate, and raffinose in UW solution [1] are more efficient in preventing the programmed cell death triggered by cell swelling during hypothermic storage as compared to the impermeants used in other solutions (e.g., glucose used in EC solution), making it an important solution for pancreas preservation [34]. We first used INS-1 cells as a model to explore a mechanism of pancreatic cell death during cold preservation. We showed that the viability of INS-1 cells is inversely related to the cell size after cold preservation, and UW solution is effective in inhibiting INS-1 cell swelling and maintaining the cell viability during cold preservation. The supplementation of DAFP-1 to UW solution can further enhance the effectiveness of UW solution in the hypothermic preservation of INS-1 cells at 4 °C, and the presence of DAFP-1 even at a submilligram level can effectively suppress the cell swelling during the cold preservation in UW solution. Among these concentrations, DAFP-1 at 500 µg/mL was determined to be an optimal concentration in combination with UW solution for the cold preservation of INS-1 cells for 72 h. Moreover, an isolated pancreatic islet containing a cluster of 2000–3000 cells was utilized to investigate whether the cold preservation of primary pancreatic islet cells would benefit from a low level of DAFP-1 in UW solution. Synergistic effects of UW solution with low levels of DAFP-1 were further evaluated with isolated rat islets toward potential tissue preservation. The cytoprotective effect of DAFP-1 at 500 µg/mL during cold preservation was further validated with the primary pancreatic islet cells. These results demonstrate that DAFP-1 at a submilligram level enhances the effectiveness of UW solution in cold preservation.

The cytoprotection provided by AFPs during hypothermic preservation is generally considered through their interactions with cell membranes preventing cold injuries [44,45,46]. To effectively stabilize cell membranes from leakage, at least a couple of milligrams of AFPs in a milliliter of solution are required for about an hour of cold storage [45]. Another study showed that TmAFP at 1.5 mM (or more than 10 mg/mL) improved the survival rate of rat insulinoma (RIN-5F) cells that were preserved at 4 °C in UW solution and suggested that TmAFP preserves the cell membrane integrity through its binding to the clathrate waters around the lipid bilayer [47]. Interestingly, our results show that TmAFP at 1 mg/mL or less has minimal improvement in the cell survival rate during the cold preservation in UW solution, suggesting that the role of TmAFP in stabilizing the cell membrane may not be apparent at such a low concentration.

**Figure 6:**
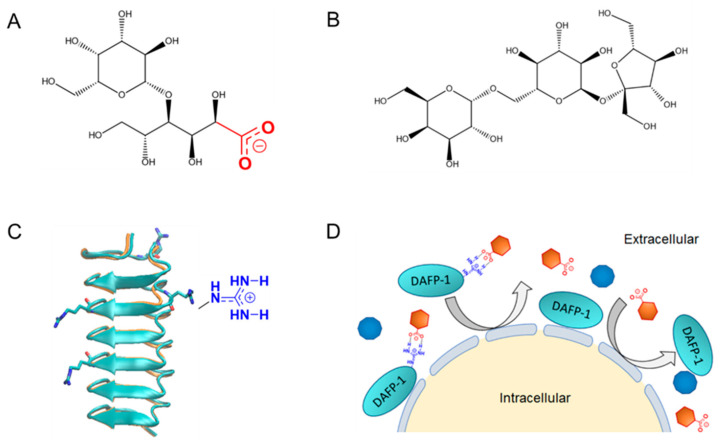
Structures and a schematic diagram for the enhancement effect. (**A**) Structure of raffinose with carboxylate group in red, (**B**) structure of lactobionate, and (**C**) structural overlay of DAFP-1 (cyan) and TmAFP (yellow–orange). The arginine residues in DAFP-1 are shown in licorice representation and colored by element (C in cyan, N in blue, O in red, and H removed for clarity). The guanidinium group (shown in blue) of an arginine residue is represented in a large aside of the protein’s structure. (**D**) Schematic representation of DAFP-1 enhancing the function of cell membrane impermeant compounds in UW solution, such as lactobionate (represented as negatively charged hexagons in orange) and raffinose (represented as decagons in blue).

To explain the perplex effects by DAFP-1 and TmAFP, the structures of the two proteins were examined closely. Arginine has been reported to be crucial in interacting with polycarboxylate and polyhydroxyl compounds in DAFP-1 for its enhancing function [26]. Interestingly, several key components in UW solution, such as lactobionate and raffinose (Figure 6A,B), belong to the classes of compounds that have been identified to effectively enhance the function of DAFP-1 primarily through their interactions with the guanidinium group of arginine in DAFP-1 (Supplemental Figure S3 and Figure 6C) [27,28,48]. The resulting complexes of these lower molecular mass molecules (referred to as enhancer molecules) and DAFP-1 would be larger than each of the molecules in size, and the interactions between these enhancer molecules and DAFP-1 can further enhance the function of DAFP-1 in preventing the freezing of water [25,27,28]. At significant levels in UW solution, lactobionate and raffinose play an important role in suppressing hypothermia-induced cell swelling. We thus postulate that DAFP-1 enhances the effectiveness of UW solution in preventing hypothermia-induced cell swelling and the consequent cell death through interacting with the cell membrane impermeants in UW solution (Figure 6D). Since lactobionate is an anion containing both carboxylate and hydroxyl groups and has a much higher concentration (100 mM) than raffinose (30 mM) in UW solution, the interactions between DAFP-1 and lactobionate would be more significant than those between DAFP-1 and raffinose.

In contrast, TmAFP does not have arginine (Supplemental Figure S3 and Figure 6C), the key residue involved in recognizing lactobionate or raffinose [26,28], which limits its interactions with these important components in UW solution. Nevertheless, possible contributions of other different residues between DAFP-1 and TmAFP as shown in their sequence alignment (Supplemental Figure S3) cannot be completely ruled out at this point. Furthermore, the cell membrane protective effect of an AFP at 1 mg/mL or lower would be ineffective [45]. Consequently, the observation of fewer improvements for the UW solution supplemented with TmAFP can be explained.

The customization of an existing preservation (or storage) solution may lead to enhancing the preservation outcome further. We expect that the effect of hypothermic protection, when DAFP-1 is used as a solution component, may be further enhanced by the addition of trehalose and other AFP enhancer molecules (e.g., polycarboxylates, polyols) [24,48]. The synergistic effect between the AFPs and other components in the cold preservation solution may also greatly lower the effective amount of the AFP in the solution, which is beneficial to the development of a cost-effective preservation solution and avoiding or minimizing potential toxic effects caused by the use of AFPs [49]. Other factors in the modification of components in the preservation solution, such as the osmotic and ion balances, should also be carefully considered and adjusted to be optimal. 

## 5. Conclusions

In summary, our study demonstrated that at a submilligram level, DAFP-1, a hyperactive beetle AFP, can enhance the effectiveness of UW solution in cold-preserving INS-1 cells, further suppress cell swelling during hypothermic preservation where cell viability is inversely related to cell size after cold preservation, and reduce hypothermia-mediated cell apoptosis. DAFP-1′s cytoprotective effect at a low level was further validated with rat islet cells during cold preservation in UW solution. By taking recently discovered synergistic effects between DAFP-1 and certain co-solutes into account, potential molecular mechanisms of a low level of DAFP-1-mediated cytoprotection during hypothermic preservation in UW solution was also discussed. We propose that the effectiveness of a cold preservation solution relies not only on the individual roles of its key components but also on the interactions involved with its key solution components. Additional studies will further evaluate the effects of AFPs supplemented preservation solutions in optimizing cell and tissue preservation for potential use in biomedical research and treatment.

## Data Availability

The data are available upon requests.

## Supplementary Materials

The following supporting information can be downloaded at: https://www.mdpi.com/article/10.3390/biom12111584/s1, Figure S1: Correlation between the cell size and viability after cold preservation of INS-1 cells in different preservation solutions; Figure S2: DAFP-1 toxicity testing in INS-1 cell culture; Figure S3: Sequence alignment of DAFP-1 and TmAFP.
