# Supplementary material for: Submilligram Level of Beetle Antifreeze Proteins Minimize Cold-Induced Cell Swelling and Promote Cell Survival
Source: Biomolecules. 2022 Oct 28;12(11):1584. doi: 10.3390/biom12111584 (PMC9687565; doi:10.3390/biom12111584)
Supplement: Supplementary file 1 [file biomolecules-12-01584-s001.zip › biomolecules-1956906-supplementary.pdf]

SUPPLEMENTAL FIGURES

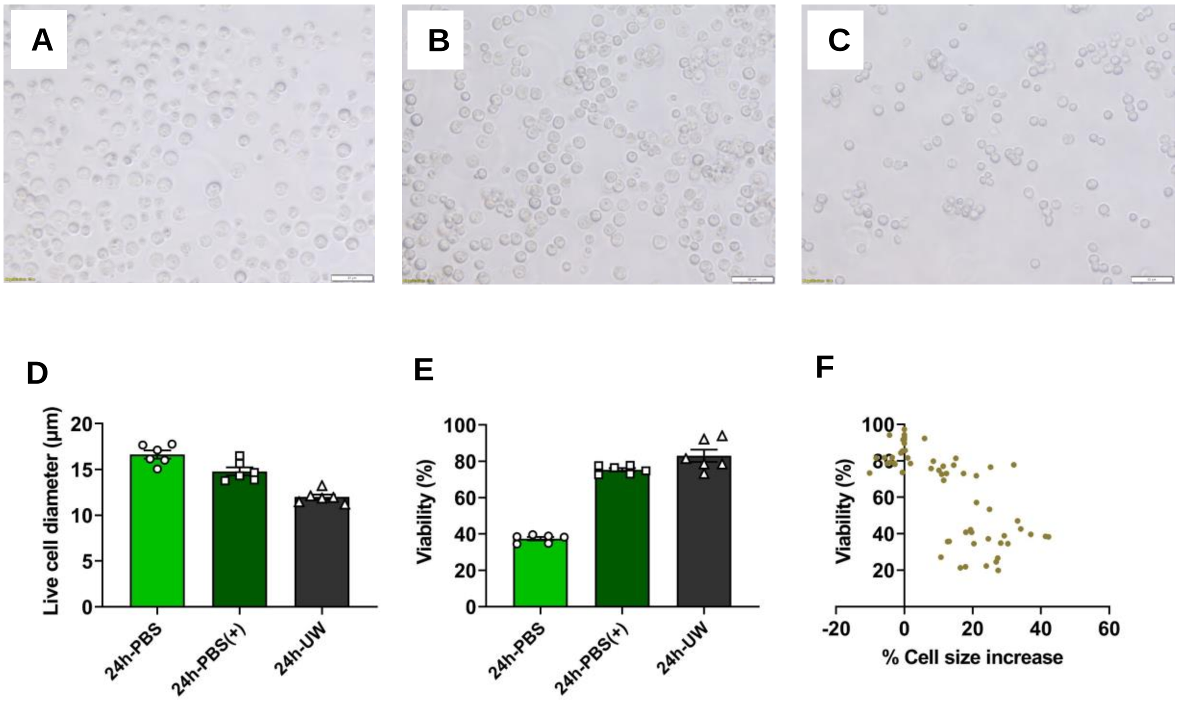

Supplemental Figure S1: Correlation between the cell size change and viability after cold preservation of INS-1 cells in different preservation solutions. INS-1 were cold preserved in PBS, PBS containing Ca^{2+}, Mg^{2+} [labeled as PBS(+)], or UW solution for up to 72 hours, and the viability and the mean diameter of live cells were assessed by trypan blue staining measured by Cellometer Auto T4. The representative morphological images of 24h-cold preserved INS-1 cells in **A)** PBS, **B)** PBS (+), and **C)** UW solution (bar=50µm). **D)** The mean diameter of the live cells and **E)** viability before and after 24h-cold preservation in PBS, PBS (+), or UW solution (n=2 in triplicates). **F)** The percent cell size increase was calculated as the difference of the live cell mean diameter between the post-preservation and pre-preservation divided by the live cell mean diameter of pre-preservation. All the viability and cell size increase measurements in each solution group from 0h, 24h, 48h, and 72h time points were used to determine the correlation between the viability and cold preserved live cell size change.

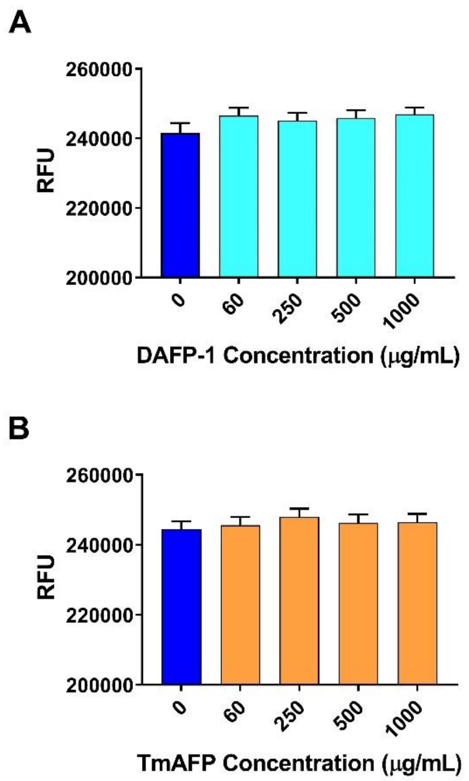

Supplemental Figure S2: Toxicity testing for DAFP-1 and TmAFP in INS-1 cell culture. INS-1 cells were cultured at 37 ºC **A)** with or without DAFP-1 (0, 60, 250, 500, and 1000 μg/mL) for 24 hours, and **B)** with or without TmAFP (0, 60, 250, 500, and 1000 μg/mL) for 24 hours. The viability was tested by the CellTiter Blue assay and shown as relative fluorescence unit (RFU). Data show the mean ± standard error of the three independent experiments.

```
DAFP-1   1   QCTGGSDCRSCTVSCTDCQNCPNARTACTRSSNCINALTCTDSYDCHNAETCTRSTNCYKAKTCTGSTNCYEATACTDST   80
             ||||| || |||  || | ||||| | || |  |  | ||| | ||  | ||| |  |  | ||| ||||| ||||| |
TmAFP    1   QCTGGADCTSCTGACTGCGNCPNAVT-CTNSQHCVKANTCTGSTDCNTAQTCTNSKDCFEANTCTDSTNCYKATACTNSS   79
DAFP-1 81   GCP--   83
             |||
TmAFP   80   GCPGH   84
```

Supplemental Figure S3: Sequence alignment of DAFP-1 and TmAFP. The arginine residues are shown in red.
